# Timing of thyroid ultrasonography in the etiological investigation of congenital hypothyroidism

**DOI:** 10.1590/2359-3997000000239

**Published:** 2017-01-27

**Authors:** Maria de Fátima Borges, Nathalie de Almeida Sedassari, Anelise de Almeida Sedassari, Luis Ronan Marquez Ferreira de Souza, Beatriz Pires Ferreira, Beatriz Hallal Jorge Lara, Heloísa Marcelina Cunha Palhares

**Affiliations:** 1 Universidade Federal do Triângulo Mineiro Uberaba MG Brasil Disciplina de Endocrinologia, Universidade Federal do Triângulo Mineiro (UFTM), Uberaba, MG, Brasil; 2 UFTM Uberaba MG Brasil Disciplina de Radiologia e Diagnóstico por Imagem, UFTM, Uberaba, MG, Brasil

**Keywords:** Congenital hypothyroidism, thyroid dysgenesis, thyroid ectopia, dyshormonogenesis, thyroid ultrasound

## Abstract

**Objectives:**

To describe the findings of thyroid ultrasonography (T-US), its contribution to diagnose congenital hypothyroidism (CH) and the best time to perform it.

**Subjects and methods:**

Forty-four patients with CH were invited to undergo T-US and 41 accepted. Age ranged from 2 months to 45 years; 23 patients were females. All were treated with L-thyroxine; 16 had previously undergone scintigraphy and 30 had previous T-US, which were compared to current ones.

**Results:**

At the current T-US, the thyroid gland was not visualized in its normal topography in 10 patients (24.5%); 31 T-US showed topic thyroid, 17 with normal or increased volume due to probable dyshormonogenesis, 13 cases of hypoplasia and one case of left-lobe hemiagenesis. One patient had decreased volume due to central hypothyroidism. Scintigraphy scans performed 3-4 years earlier showed 100% agreement with current results. Comparisons with previous T-US showed concordant results regarding thyroid location, but a decrease in current volume was observed in eight due to the use of L-thyroxine, calling the diagnosis of hypoplasia into question.

**Conclusions:**

The role of T-US goes beyond complementing scintigraphy results. It allows inferring the etiology of CH, but it must be performed in the first months of life. An accurate diagnosis of CH will be attained with molecular study and the T-US can guide this early assessment, without therapy withdrawal.

## INTRODUCTION

In the early years of life, thyroid ultrasonography (T-US) can be performed on individuals with suspected congenital hypothyroidism (CH), assisting in the etiological diagnosis. Thyroid dysgenesis includes: athyrosis, as an “empty” thyroid area with or without ectopic tissue (agenesis), and thyroid hypoplasia. When the thyroid, visualized at the ultrasound assessment, has normal or increased volume, one of several forms of dyshormonogenesis is suggested (1,2).

The diagnosis of CH through screening programs has greatly contributed to the start of treatment within an adequate time frame and the etiological diagnosis has been sought by these programs aiming at genetic counseling (3-7).

Due to the need to initiate treatment within the first month after birth aiming to preserve the child’s neuronal development, the investigation of CH etiology is usually delayed until the age of 3, when treatment withdrawal for one month does not result in much impairment or, in many cases, is not performed due to the lack of resources in the region where the child resides or due to limited mobility to the capital cities, which are the centers of screening programs (6,7).

The aim of the present study was to describe the ultrasonographic findings and their contribution to the diagnosis of CH in a series of 41 patients, as well as evaluate the most appropriate time to perform the T-US.

## SUBJECTS AND METHODS

Forty-four individuals with a previous diagnosis of CH were invited to undergo T-US at the Imaging Service of *Universidade Federal do Triângulo Mineiro* (UFTM). Of these patients, 23 were from the State’s Newborn Screening Program (SNSP) and were being followed at the Municipal Health Unit of Uberaba, while another 21 patients being followed at the Department of Endocrinology of UFTM were from Uberaba and other municipalities in the Triangulo Mineiro region, state of Minas Gerais, Brazil. A total of 41 patients, 23 females and 18 males, aged 0.2 to 45 years (median: 6 years) accepted the invitation to undergo T-US. The study was approved by the local University Medical Ethic Committee.

All patients were undergoing treatment since the diagnosis, with the exception of one patient who had a positive newborn screening test, but whose TSH levels normalized after medication withdrawal, being considered a case of transient hyperthyrotropinemia (case 21).

Of the patients referred by the SNSP (n: 23), 15 had already undergone etiological investigation after treatment withdrawal between 3 and 4 years old, having been submitted to T-US and scintigraphy. Of the patients from UFTM (n: 21), 15 had undergone T-US and of these, only two had undergone scintigraphy. Therefore, of the total of 41 patients, 30 had undergone T-US and 17 scintigraphy, prior to the study (Tables 1 and 2).

**Table 1:** Data from congenital hypothyroidism patients whose thyroids were not visualized on Ultrasonography (T-US) or with ectopic thyroid confirmed by scintigraphy (131I)

Case N.	Gender	Initial T-US		TT	Current T-US		TT	^131^I	Diagnosis
	
		Local	Total volume (mL)		Local	Total volume (mL)			
1	F	E	0.81	3	E	-	7.0	SME	Ectopic
2	M	NV	-	3	E	-	4.4	SLE	Ectopic
3	M	NV	-	4	NV	-	3.6	-	Dysgenesis
4	M	T	1.0	10	NV	-	15.7	-	Dysgenesis
5	M	T	1.2	29	E	-	32.0	SLE	Ectopic
6	M	NV	-	19	NV	-	21.0	Agenesis	Dysgenesis
7	F	NV	-	14	NV	-	20.8	-	Dysgenesis
8	F	NV	-	3	NV	-	14.0	-	Dysgenesis
9	M	-	-	-	NV	-	0.7	-	Dysgenesis
10	M	-	-	-	NV	-	2.2	-	Dysgenesis

TT: time of treatment (years); E: ectopic; SME: submentonian ectopia; NV: not visualized; SLE: sublingual ectopia; T: topic.

Of the 30 patients with previous T-US, test results found in the medical records of 23 patients allowed a comparison with the current results, as they contained thyroid descriptions and volumes (Tables 1 and 2).

**Table 2:** Sonographic (T-US) and scintigraphic (131I) data of congenital hypothyroidism patients with topic thyroids and etiological diagnosis

Case N.	Gender	Initial T-us		TT	Current T-uS		TT	^131^I	Diagnosis
	
		Local	Total volume (mL)		Local	Total volume (mL)			
11	F	T	1.85	3.0	T	LLH	8.1	T	LLH
12	M	T	4.34*	3.0	T	0.70*	9.0	T	D
13	F	T	4.35*	3.0	T	2.80*	9.0	T	D
14	F	T	0.26	4.0	T	0.20	4.7	-	H
15	F	T	0.73	3.5	T	1.00	6.0	T	H
16	F	T	1.79*	3.0	T	1.20*	8.5	T	D
17	F	-	-	-	T	2.50	4.0	T	D
18	M	T	0.50	4.0	T	0.80	4.6	-	H
19	F	T	0.89	3.5	T	1.00	7.9	T	H
20	M	T	13.2*	3.0	T	6.50*	10.8	T	D
21	F	-	-	-	T	3.00	3.0	T	TH
22	F	T	1.41	3.0	T	1.50	6.9	T	D
23	M	T	Not described	3.0	T	14.20	14.0	T	D
24	M	T	2.30*	3.0	T	0.50*	14.4	T	HC
25	F	-	-	-	T	1.10	6.0	-	H
26	F	T	0.94	7.0	T	3.00	11.0	-	D
27	M	T	1.70	0.1	T	2.10	5.9	-	D
28	F	T	0.50	0.1	T	0.85	12.5	-	H
29	F	T	0.90	1.0	T	1.00	4.9	-	D
30	M	T	7.70	8.0	T	8.50	13.0	-	D
31	F	-	-	-	T	4.50	1.8	-	D
32	M	T	11.00*	0.1	T	3.00*	2.0	-	D
33	F	T	5.40*	37.0	T	2.00*	45.0	-	D
34	F	T	1.90	5.0	T	2.00	20.0	-	D
35	M	T	2.35	2.0	T	2.40	11.4	-	D
36	F	-	-	-	T	5.50	6.0	-	D
37	F	-	-	-	T	0.20	0.2	-	H
38	F	-	-	-	T	1.90	1.5	-	D
39	M	T	13.00*	0.1	T	3.00*	0.5	-	D
40	M	-	-	-	T	1.90	1.0	-	D
41	F	-	-	-	T	0.80	0.9	-	D

TT: time of treatment (years); T: topic; LLH: left lobe hemiagenesis; D: dyshormonogenesis; H: hypoplasia; TH: transient hyperthyrotropinemia; CH: central hypothyroidism. * Decrease in volume from previous to current T-US.

All children participating in the study underwent T-US, which evaluated the following thyroid gland characteristics: position, texture, volume and additional findings. The examinations were performed with patients in the supine position with neck hyperextension (1). The assessment started with the cervical region evaluation from the base of the tongue to the thyroid, characterizing its lobes and the isthmus regarding their depth, length and width, evaluating the neck position, echotexture, calculated dimensions and volumes according to the formula π/6 x depth x length x width (8). Total thyroid volume was obtained by adding both lobes, whereas the isthmus was disregarded when determining the volume. Color Doppler was also used to assess its behavior. If any thyroid lesion was identified, it was recorded and analyzed separately.

The ultrasound assessments were performed in the Radiology and Imaging Diagnostic Department of Hospital de Clinicas, UFTM. The ultrasound equipment used for the assessments was a Philips device, model HD11 (Philips Healthcare, The Netherlands) with a 7.5-MHz frequency linear transducer (3-13 MHz). An Accuvix V10 device (Samsung Medison Heathcare) with a 7.5 MHz frequency linear transducer (6-12 MHz) was also used.

Reference values used for individuals aged ≥ 6 years to evaluate the volumes obtained were the ones described by Chanoine and cols. (9), and Zimmermann and cols. (10), i.e., thyroids with a total volume < 1.5 mL were considered compatible with low volume and, therefore, hypoplastic. In individuals aged up to 5 years, the reference values used were those obtained at the service itself from 30 normal children and stratified by age group, as follows: from 2 months to 3 years (n: 15) ranging from 0.52 to 1.3 mL; and from 3.1 to 5 years (n: 15) ranging from 0.98 to 2.7 mL. In adults, the volume considered normal was 10.3 ± 5.1 mL (11).

### Statistical analysis

Numerical data are shown as median, minimum and maximum values. Student’s *t* test was employed when comparing volumes obtained at prior x current T-US, with P < 0.05 being considered significant.

## RESULTS

Of the 44 patients invited to participate in the study, 41 underwent T-US. The thyroid gland was not visualized in its bed in 10 cases (24.5%), which were considered as thyroid dysgenesis (Table 1). Of these, four had undergone scintigraphy, of which three had an ectopic thyroid and one thyroid agenesis. In this group, eight patients had previously undergone T-US. In case 1 the thyroid was located in the submentonian region, which was confirmed by scintigraphy. In two cases (cases 4 and 5) the thyroid was topic, but was not seen in the current T-US. In case 4, the thyroid was very small and its volume might have reduced even further with L-thyroxine treatment, but in case 5, the thyroid gland was located in the sub-lingual region at the scintigraphy, showing a disagreement between the initial T-US and the scintigraphy (Table 1).

Thirty-one patients had topic thyroids (75.6%), with volumes ranging from 0.2 to 14.2 mL (median: 1.95 mL). One patient had left-lobe hemiagenesis, confirmed by a previous T-US. Thirteen patients (cases 12, 14, 15, 16, 18, 19, 22, 24, 25, 28, 33, 34 and 37) had homogeneous thyroids with reduced volumes for their ages, which could be considered hypoplastic if they had not undergone a previous T-US for comparison.

In this group, 11 had undergone previous T-US and in six, thyroid volume was normal for age. Of these six patients, four showed a reduction in volume over the course of treatment with L-thyroxine (cases 12, 16, 24 and 33) and case 14 was diagnosed with central hypothyroidism. Thus, taking into account these particularities, only seven of the 13 patients could be actually considered as having true hypoplasia and, therefore, thyroid dysgenesis, being one patient with central hypothyroidism and the remaining five with dyshormonogenesis (Table 2).

As for the other cases of topic thyroid (n = 17) (Table 2), the patients showed normal or increased thyroid volumes according to the reference standards for age. In this group, 11 had previously undergone T-US, confirming the diagnosis of dyshormonogenesis. It was also observed that four cases had a reduction in thyroid volume with treatment, but still kept within normal limits for age.

**Figure 1:**
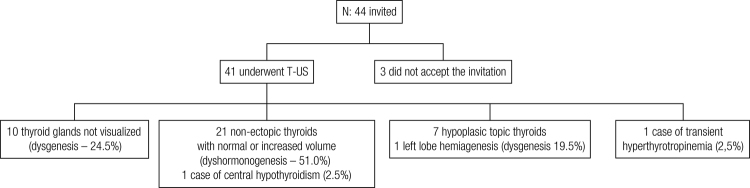
Patient distribution according to thyroid location after ultrasonography and etiological diagnosis.

Thus, of the total of 31 patients, one case of thyroid hemiagenesis, seven cases of true hypoplasia, one case of central hypothyroidism, one of transient hyperthyrotropinemia (21 cases) and 21 cases of dyshormonogenesis were identified (Figure 1).

In the group of topic thyroid, scintigraphy with ^123^I had been performed on 12 patients, and showed 100% agreement with the results obtained from the current T-US, considering the thyroid location. When analyzing the two groups (topic and ectopic thyroid), scintigraphy showed to be more accurate in locating the remaining ectopic gland. It also allowed identifying the iodine organification defect in three cases through the perchlorate test performed in 11 patients with topic thyroid. The remaining patients did not undergo scintigraphy due to younger ages or because they did not have access to the exam.

## DISCUSSION

A child with a confirmed diagnosis of CH needs prompt treatment with L-thyroxine and the etiological research may be delayed until 3 years of age, considering that the first concern is to preserve the child’s developing central nervous system, growth and cognitive capacity (12-16).

Some doctors advise parents that, at some point, the drug will be discontinued and the etiology of CH will be investigated (6,7). In some cases, this information generates a degree of anxiety and an indeterminate diagnosis can lead to difficulties in the relationship between doctor and parents, who also inquire about treatment duration. The physician should convey confidence and reassure the parents that there is a best moment for this investigation to occur.

In clinical practice, the institution of L-thyroxine treatment in a timely manner was a breakthrough of the screening programs and, until recently, there were no resources for the study of CH etiology, considered a research subject limited to only a few centers of excellence and this is not the case in many Brazilian states (3-7).

This series includes some children who were investigated between 3 and 4 years of age, in the city of Uberaba, from SNSP-MG, but there are adults aged 45 years that received the diagnosis of CH at birth, have been using L-thyroxine adequately and the T-US was the only test they had access to. In adulthood, the patients themselves ask their doctors why they have the disease, express their desire to know the nature of the defect and whether they could pass it on to offspring, prompting the physician to attain a more accurate diagnosis to support genetic counseling.

As it is currently carried out, the etiological research of CH is difficult and inaccurate, as well as expensive and limited to large centers in the capital cities of Brazil. Thyroid scintigraphy, considered the “gold standard” for the gland identification, could be performed in the neonatal period when treatment has not been started yet, but would require prompt availability of a Nuclear Medicine Service, only available in certain cities (2). Most often, it is carried out much later in life, after medication withdrawal for at least a month (6).

The T-US complements the scintigraphy; it is very effective for thyroid identification and can be performed in the neonatal period, after treatment has been instituted (2). When the gland is located in its normal topography, the T-US can also disclose its characteristics, and according to the volume found, it may indicate whether it is normal, increased or decreased, or whether there is hemiagenesis of one of the lobes (1,17).

In the present study, we observed very good agreement between the two tests regarding thyroid location. In 10 cases the thyroid was not visualized by T-US, probably due to dysgenesis. Among the 31 cases of topic thyroid, 21 had normal or increased volume for age, probably due to dyshormonogenesis, 7 had topic thyroid with reduced volume, configuring thyroid hypoplasia, one case showed central hypothyroidism, one case had transient hyperthyrotropinemia and one case had left lobe hemiagenesis, a rare variant of dysgenesis (18). However, to attain a secure diagnosis, the T-US should be performed as soon as possible, and not much later as it was done, after long-term use of thyroid hormone (2).

In 22 cases of topic thyroid gland, thyroid volume obtained through the previous T-US was compared with the current one, demonstrating volume reduction in eight. This probably occurred due to L-thyroxine use since birth, which reduces volume and changes characteristics of the gland (19). These data indicate that the best time to perform the T-US is within the first few months after birth, when the thyroid still retains its original features and when it is topic and has reduced volume, CH may be due to hypoplasia or central hypothyroidism. A patient with central hypothyroidism (case N. 14) had decreased thyroid volume, decreased TSH with low free T4 and a prior investigation had revealed a TSH-receptor defect (20,21). Increased thyroid volume was also observed in some cases, which can be explained by the fact that thyroid volume increases with age, especially after 8 years old (22).

A T-US limitation is that when the thyroid is ectopic, its location is not always defined, in which case scintigraphy is superior, being effective in accurately locate the gland along the embryonic migration path (17). In a previous study involving a few cases of this series (6) scintigraphy effectively located two ectopic thyroids in 15 cases and when the perchlorate test was performed, it indicated iodine organification defect in three cases. In other cases of dyshormonogenesis, scintigraphy associated with the perchlorate test, as well as the T-US do not have the power to diagnose the cause of dyshormonogenesis.

Further progress toward etiological diagnosis will come with molecular studies of congenital thyroid defects (23-25) and the T-US can direct the initial evaluation, without requiring therapy interruption. The collection of material for DNA extraction can be carried out by a small laboratory and the material may be sent to referral centers closer to the municipality where the child resides.

Until recently, thyroid dysgenesis was considered a sporadic event (26). In recent years, there have been reports of families with multiple affected cases and molecular biology studies have demonstrated the involvement of genes (*TTF1* and *2, PAX8, TSH-R*), which encode highly conserved transcription factors that result in agenesis, ectopia and thyroid hypoplasia, when inactivated (27,28).

Furthermore, the description of other affected genes responsible for cases of dyshormonogenesis (25) will make the etiological diagnosis much more practical and objective. Currently, the main proteins responsible for most of the critical biochemical steps in thyroid hormone synthesis have been identified, with increasing knowledge of the phenotype/genotype correlation, allowing a more accurate diagnosis to be attained (25). Even the transient hyperthyrotropinemia diagnosed in one of our cases can interfere with cognitive development and subsequently express as manifest hypothyroidism and has been considered a thyroid hormonogenesis defect (29).

We conclude that the role of T-US is more than a complementary test to scintigraphy. Its early performance, after the diagnosis of CH, in the first years of life allows suggesting the diagnosis of hypoplasia or dyshormonogenesis according to the observed volume and characteristics and, when the gland is not visualized, it allows suggesting thyroid dysgenesis. According to this classification patients may be referred to molecular study of thyroid dysgenesis or dyshormonogenesis in the first year of life, without the need for treatment interruption, which offers more comfort to the patient, allowing the physician to attain an accurate diagnosis and offer adequate genetic counseling.

## References

[1] Garel C, Léger J. Thyroid imaging in children. Endocr Dev Basel. 2007;10:43-61. 10.1159/000106819 17684389

[2] Léger J, Olivieri A, Donaldson M, Torresani T, Krude H, van Vliet G, et al.; ESPE-PES-SLEP-JSPE-APEG-APPES-ISPAE; Congenital Hypothyroidism Consensus Conference Group. European Society for Paediatric Endocrinology consensus guidelines on screening, diagnosis, and management of congenital hypothyroidism. J Clin Endocrinol Metab. 2014;99:363-84. 10.1210/jc.2013-1891 PMC4207909 24446653

[3] Maciel LM. Hipotireoidismo congênito. Projeto Diretrizes – Associação Médica Brasileira e Conselho Federal de Medicina. 2011. Available at: <http://www.projetodiretrizes.org.br/ans/diretrizes/hipotireoidismo_congenito.pdf>. Accessed on: Apr. 23, 2014.

[4] Pezzuti IL, Lima PP, Dias VM. Congenital hypothyroidism: the clinical profile of affected newborns identified by the Newborn Screening Program of the State of Minas Gerais, Brazil. J Pediatr (Rio J). 2009;85:72-9. 10.2223/JPED.1863 19198739

[5] Delange F. Neonatal screening for congenital hypothyroidism: results and perspectives. Horm Res. 1997;48:51-61. 10.1159/000185485 9251921

[6] Palhares HM, Silva LC, Sato LM, Lara BH, Miranzi SSC, Silva AP, et al. Incidence of congenital hypothyroidism in the city of Uberaba/Minas Gerais and etiological evaluation of the affected subjects. Arq Bras Endocrinol Metab. 2012;56:305-12. 10.1590/s0004-27302012000500005 22911283

[7] Dias VM, Campos AP, Chagas AJ, Silva RM. Congenital hypothyroidism: etiology. J Pediatr Endocrinol Metab. 2010;23:815-26. 10.1515/jpem.2010.131 21073124

[8] Brunn J, Block U, Ruf G, Bos I, Kunze WP, Scriba PC. Volumetrie der Schilddrüsenlappen mittels Riel-time-Sonographie. Dtsch Med Wochenschr. 1981;106:1338-40. 10.1055/s-2008-1070506 7274082

[9] Chanoine JP, Toppet V, Lagasse R, Spehl M, Delange F. Determination of thyroid volume by ultrasound from the neonatal period to late adolescence. Eur J Pediatr. 1991;150:395-9. 10.1007/BF02093716 2040346

[10] Zimmermann MB, Hess SY, Molinari L, Benoist B, Delange F, Braverman LE, et al. New reference values for thyroid volume by ultrasound in iodine-sufficient school children: a World Health Organization/Nutrition for Health and Development Iodine Deficiency Study Group Report. Am J ClinNutr. 2004;79:231-7. 10.1093/ajcn/79.2.231 14749228

[11] Berghout A, Endert E, Ross A, Hogerzeil HV, Smits NJ, Wiersinga WM. Thyroid function and thyroid size in normal pregnant women living in an iodine replete area. Clin Endocrinol (Oxf). 1994;41:375-9. 10.1111/j.1365-2265.1994.tb02560.x 7955445

[12] Balhara B, Misra M, Levitsky LL. Clinical monitoring guidelines for congenital hypothyroidism: laboratory outcome data in the first year of life. J Pediatr. 2011;158:532-7. 10.1016/j.jpeds.2010.10.006 21094953

[13] Klein AH, Meltzer S, Kenny FM. Improved prognosis in congenital hypothyroidism treated before age three months. J Pediatr. 1972;81:912-5. 10.1016/s0022-3476(72)80542-0 5086719

[14] Silva KA, Harper A, Downs A, Blasco PA, Lafranchi SH. Neurodevelopment outcomes in congenital hypothyroidism: comparison of initial T4 dose and time to reach target T4 and TSH. J Pediatr. 2005;147:775-80. 10.1016/j.jpeds.2005.07.024 16356430

[15] Rose SR, Brown RS, Foley T, Kaplowitz PB, Kaye CI, Sundararajan S, et al. American Academy of Pediatrics; Section on Endocrinology and Committee on Genetics, American Thyroid Association; Public Health Committee, Lawson Wilkins Pediatric Endocrine Society. Update of newborn screening and therapy for congenital hypothyroidism. Pediatrics. 2006;117:2290-303. 10.1542/peds.2006-0915 16740880

[16] LaFranchi SH. Approach to the diagnosis and treatment of neonatal hypothyroidism. J Clin Endocrinol Metab. 2011;96:2959-67. 10.1210/jc.2011-1175 21976744

[17] De Bruyn R, Ng WK, Taylor J, Campbell F, Mitton SG, Dicks-Mireaux C, et al. Neonatal hypothyroidism: comparison of radioisotope and ultrasound imaging in 54 cases. Acta Paediatr Scand. 1990;79:1194-8. 10.1111/j.1651-2227.1990.tb11409.x 1964760

[18] Castanet M, Leenhardt L, Léger J, Simon-Carre A, Lyonnet S, Pelet A, et al. Thyroid hemiagenesis is a rare variant of thyroid dysgenesis with a familial component but without Pax8 mutations in a cohort of 22 cases. Pediatr Res. 2005;57:908-13. 10.1203/01.PDR.0000161409.04177.36 15845640

[19] Bubuteishvili L, Garel C, Czernichow P, Léger J. Thyroid abnormalities by ultrasonography in neonates with congenital hypothyroidism. J Pediatr. 2003;143:759-64. 10.1067/S0022-3476(03)00537-7 14657824

[20] Biebermann H, Schöneberg T, Krude H, Schultz G, Gudermann T, Gruters A. Mutations of the human thyrotropin receptor gene causing thyroid hypoplasia and persistent congenital hypothyroidism. J Cin Endocrinol Metab. 1997;82:3471-80. 10.1210/jcem.82.10.4286 9329388

[21] Santos A, Lara BH, Palhares HM, Ferreira BP, Domené H, Scaglia PA, et al. C105fs114x thyrotropin beta-subunit gene mutation resulting in congenital central hypothyroidism: a genetic study of a Brazilian Family. Horm Res Paediatr. 2011;76: (suppl 4): abst 11.

[22] Fleury Y, Van Melle G, Woringer V, Gaillard RC, Portmann L. Sex-dependent variations and timing of thyroid growth during puberty. J Clin Endocrinol Metab. 2001;86:750-4. 10.1210/jcem.86.2.7209 11158041

[23] Macchia PE. Recent advances in understanding the molecular basis of primary congenital hypothyroidism. Mol Med Today. 2000;6:36-42. 10.1016/s1357-4310(99)01620-2 10637573

[24] Rubio IGS, Knobel M, Nascimento AC, Santos CL, Toniolo JV, Medeiros-Neto G. Hipotireoidismo congênito: recentes avanços em genética molecular. Arq Bras Endocrinol Metab. 2002;46:391-401.

[25] De Felice M, Di Lauro R. Thyroid development and its disorders: genetic and molecular mechanisms. Endocr Rev. 2004;25:722-46. 10.1210/er.2003-0028 15466939

[26] Castanet M, Polak M, Bonaïti-Pellié C, Lyonnet S, Czernichow P, Léger J. Nineteen years of national screening for congenital hypothyroidism: familial cases with thyroid disgenesis suggest the involvement of genetic factors. J Clin Endocrinol Metabolism. 2001;86:2009-14. 10.1210/jcem.86.5.7501 11344199

[27] Castanet M, Polak M, Léger J. Familial forms of thyroid dysgenesis. Endocr Dev. 2007;10:15-28. 10.1159/000106817 17684387

[28] Ramos HE, Nesi-França S, Boldarine VT, Pereira RM, Chiamolera MI, Camacho CP, et al. Clinical and molecular analysis of thyroid hypoplasia: a population-based approach in Southern Brazil. Thyroid. 2009;19:61-8. 10.1089/thy.2008.0116 18976153

[29] Moreno JC, Visser TJ. New phenotypes in thyroid dyshormonogenesis: hypothyroidism due to DUOX2 mutations. Endocr Dev. 2007;10:99-117. 10.1159/000106822 17684392

